# High-temperature quality of accelerated spheroidization on SUP9 leaf spring to enhance machinability

**DOI:** 10.1016/j.heliyon.2018.e01076

**Published:** 2018-12-26

**Authors:** Hendri Chandra, Diah Kusuma Pratiwi, Muhammad Zahir

**Affiliations:** Department of Mechanical Engineering, Sriwijaya University, 30662, Indonesia

**Keywords:** Mechanical engineering, Metallurgical engineering

## Abstract

The high temperature on the austenitization showed the varied results in the fast spheroidization in high carbon leaf spring steel SUP9/55CrMnA. The heating temperature was held for 15 minutes in the austenitization temperature at 800, 850, and 900 °C respectively, which was intended to break cementite lamellar and then proceed to spheroidization at 720 °C for 2 hours. The purpose of this treatment was to enhance machinability, the experiment results showed a significant enhancing in machinability at 800 °C with 180 VHN hardness value and machining performance increased to 78.5% with 54 seconds machining time. The drilling process used an HSS twist drilling 2.5 mm in diameter and a workpiece height of 15 mm under equal spindle speed and load. The machining process is recommended to know the quality of the cementite, which was revealed on the drilling process of the initial specimen, the drill bit was unable to drill the specimen caused by chisel edge blunted. The acceleration spheroidization process under high temperature showed the significant enhancing in machinability of SUP9 leaf spring.

## 1 Introduction

The spheroidization is a process that aims to increase the machinability of the material. The main problem of the spheroidization process is time-consuming in holding time in order to break lamellar cementite into a spheroidal. So many researches have been carried out to shorten the process of the holding time. The holding time of the spheroidization process is normally maintained at below the $A_{e1}$ temperature, the particle of carbide in the ferrite matrix show a high ductility and has a lower hardness [1].

The spheroidization process can be defined as two stages i.e. breaking cementite lamellar and coarsening cementite particles [2]. Saha et al. [3] and Lv et al. [4] has investigated the cyclic heat treatment on the spheroidization process, but has been found the distinction of defining the quality of the austenitization temperature and holding time on these treatment comparisons. Saha et al. [3] used 8-cycle in 810 °C with holding time for 6 min, followed by forced air cooling, with a homogenizing annealed 0.6% C steel in 1100 °C for 60 min. The best result was achieved after 8-cycles and bad result at 5-cycles. Otherwise, Lv et al. [4] on the investigation showed the good result at 5-cycles and strain decrease at 7-cycles, from the comparison of two investigations that how many numbers of the cyclic has still been a problem. This investigation tries to determine the equivalent of high-quality temperatures and suitable holding time in the fast spheroidization process.

The complexity of the fast spheroidization process is to determine the value of high temperature and suitable holding time. The result of this condition will generate steel with fine cementite lamellar (decrease hardness) or generate carbide precipitation intermetallic compound (increase hardness) [5].

The machinability aims to increase performance on the material removal from influence by workpiece properties. The annealing process is used for enhancing machinability in medium carbon alloys, but spheroidization is used to obtain lower hardness structure and increase machinability in high carbon alloys [6]. The machinability conditions are measured by a wide variety of machining process that involves the cutting ability, cutting process, and surface quality. The high carbon steel workpiece is having high hardness value that contains cemented carbide, which is unfavourable on tool life caused by the influence of cutting edge when breaking cementite lamellar. Cementite is the hardest and brittle properties of metallographic constituent [7].

The advantages of brittle properties are achieved low built-up edge (BUE), which is good for surface quality and otherwise, the hardest properties of cementite decrease the machining performance caused by increased cutting force, tool wear, and temperature. The heat treatment is conducted to achieve the material according to our demand, where the fast spheroidization process is conducted to enhancing machinability.

## 2 Experimental

The investigation was performed to the high carbon steel from leaf spring steel 55CrMnA or usually called as SUP9, with material composition, 0.762%Cr - 0.884%Mn - equivalent 0.655%C (wt.%) that obtained from metal analysis testing Niton XL2, for the mechanical and thermal properties of SUP9 material able to be shown in Table 1. In this investigation, the specimen size was used with dimensions 60 mm length, 25 mm width, and 15 mm thick.

**Table 1:** Mechanical and thermal properties of SUP9 material.

**Mechanical Properties**	**Elastic Modulus (GPa)**	**Poisson Ratio**	**Tensile Strength (MPa)**	**Yield Strength (MPa)**	**Shear Modulus (GPa)**
	190	0.29	1225	1080	73
**Thermal Properties**	**Melting Point (°C)**	**Specific Heat Capacity (J/kg-K)**		**Thermal Conductivity (W/m-K)**	**Thermal Expansion (μm/m-K)**
	1450	470		48	13

**Fig. 1:**
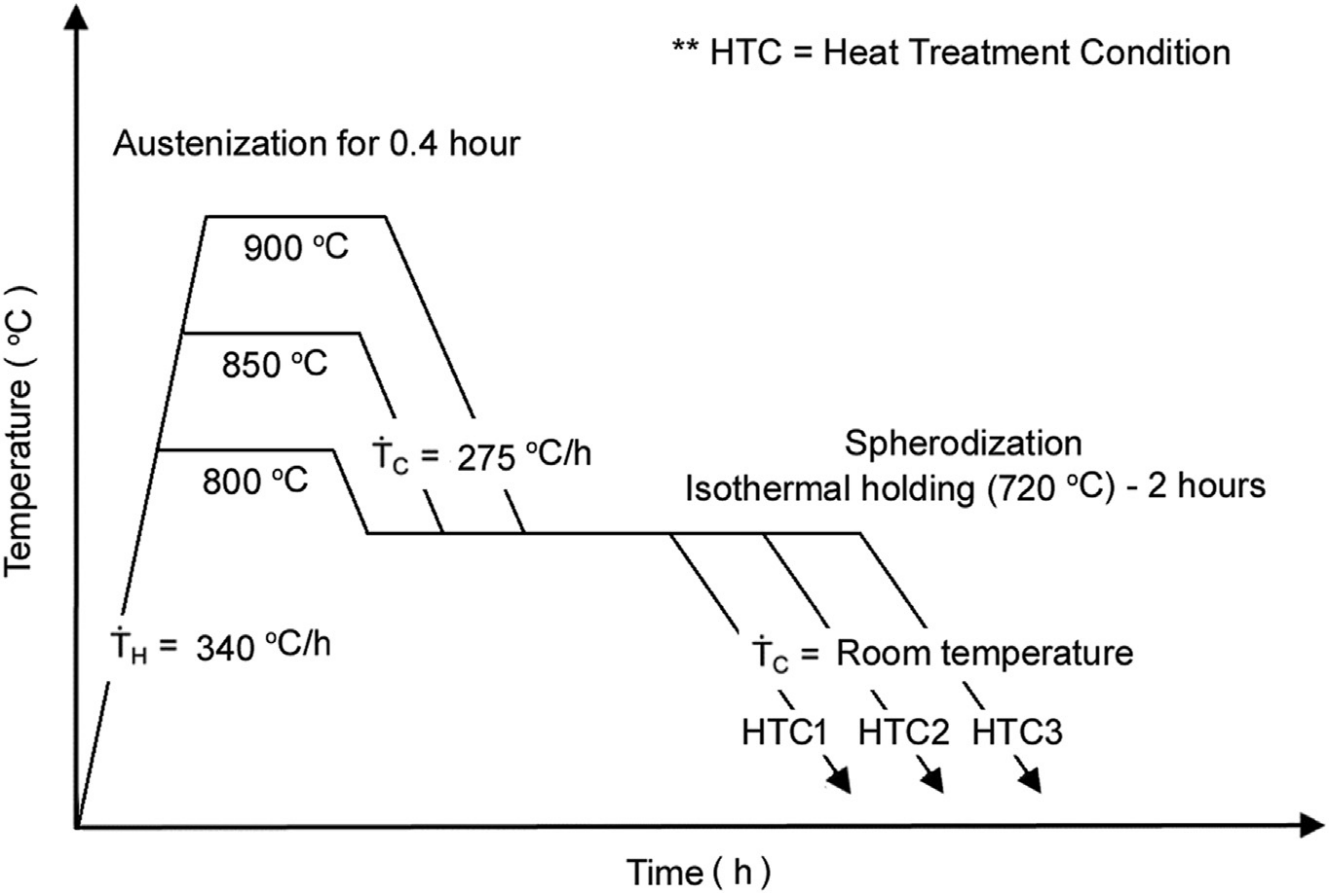
Heat treatment condition of the spheroidization SUP9.

The three heat-treatment condition (HTC) was used in this process, i.e. the condition in austenitization temperature at 800 (HTC1), 850 (HTC2), and 900 °C (HTC3) respectively, which aims to determine the quality of austenitization temperature in breaking cementite lamellar. The austenitization temperature was held for 15 minutes, furthermore continued by the spheroidization process at 720 °C with 2 hours holding time and cooling at room temperature. The heating speed in the furnace at 340 °C/h and the furnace temperature decrease in speed at 275 °C/h, as the process able to be shown in Fig. 1.

After the heat treatment process, it will be continued into microstructure observations were carried out using optical microscope under etching in 4% of Nital. The cold working the machining process was used as the procedure in determine the machinability, where the specimens will be drilled on the upright drilling conventional machine without lubrication (dry machining) with equal spindle speed and load, where its performance value will be reported to the drill ratio value when drilling the specimen (cutting ability).

**Fig. 2:**
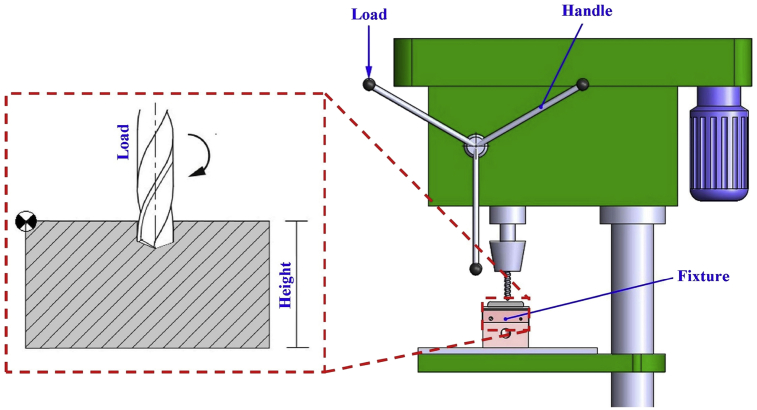
The parameter of drilling process with equal spindle speed and load.

The drill bit with a diameter of 2.5 mm was drilled on the specimen with height of 15 mm, approx. 500 rpm of spindle speed, and the load with weight approx. 3 kg was placed on the handle that regulate feeding value automatically as shown in (Fig. 2). The drilling process aims to know the quality of spheroidal cementite that occurs after the spheroidization process, which will affect the performance of machining process.

**Fig. 3:**
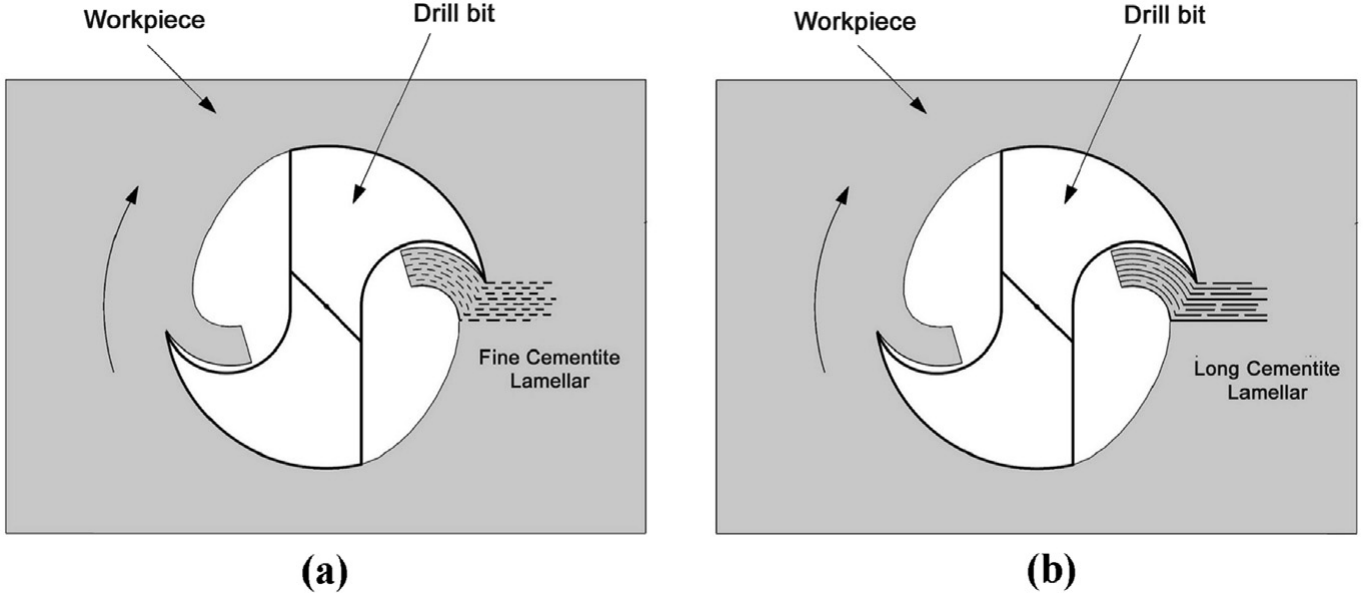
The influence of cutting edge on the cementite lamellar quality.

The cementite quality will affect in tool edge, where it is able to be wear, decrease tool life, and poor surface quality if the cutting process in the long cementite lamellar condition, as shown in Fig. 3 (b). Otherwise, in Fig. 3 (a) is depicted the condition good cutting process will be achieved if the fine cementite lamellar able to be obtained from heat treatment of spheroidization.

## 3 Results and discussion

The temperature variations at austenitization showed significant results; it was shown from the value of hardness and machining performance. From hardness test showed that HTC1 (800 °C) reached a significant value of 180 VHN, while for HTC2 (850 °C) and HTC3 (900 °C) showed hardness number 228 and 286 VHN respectively as shown in Table 2.

**Table 2:** The value Vickers hardness testing.

Vickers Hardness Number			
Initial specimen	HTC1 (800 °C)	HTC2 (850 °C)	HTC3 (900 °C)
412	180	228	286

**Fig. 4:**
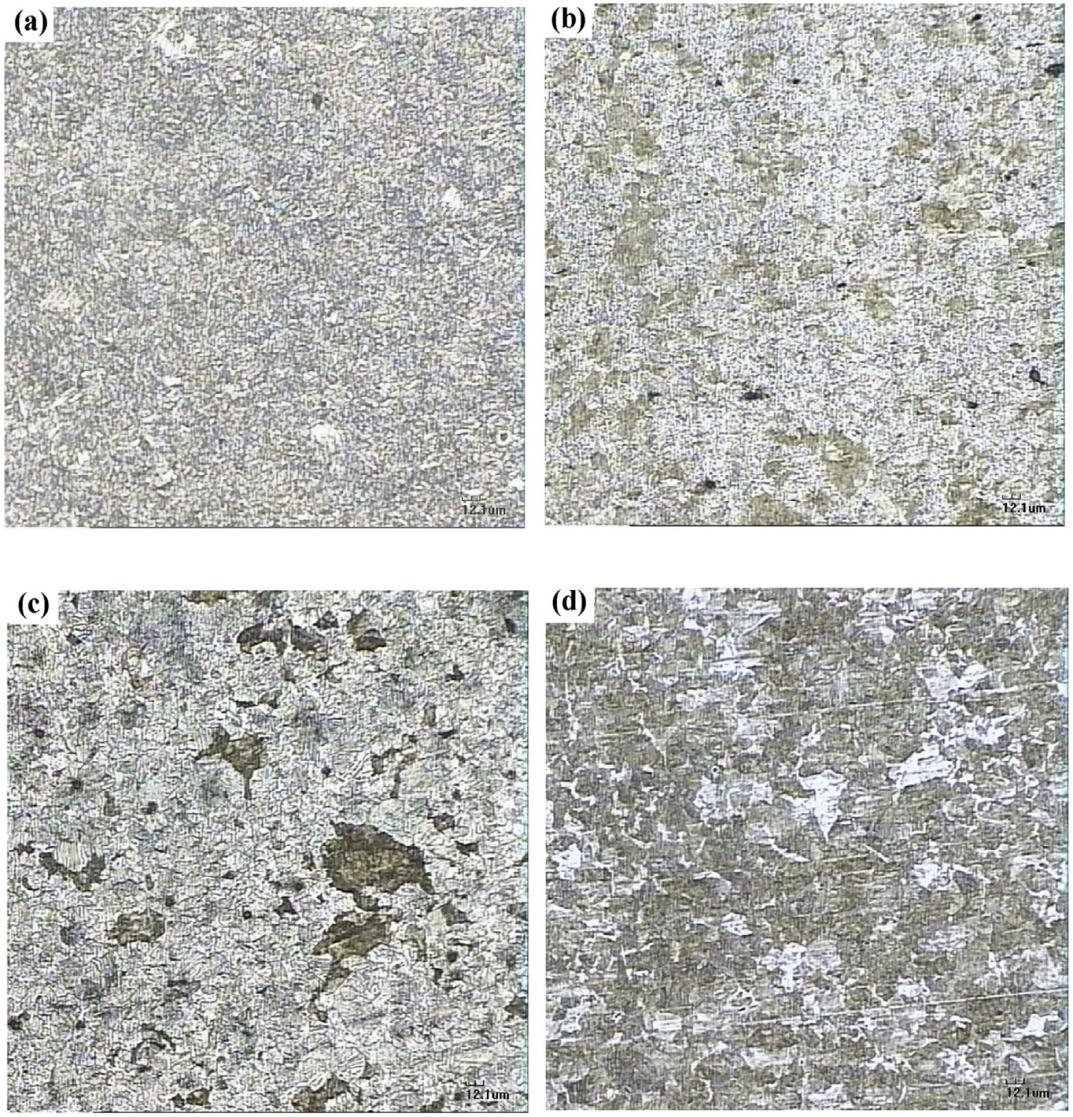
The optical microscopy results with 200x magnification of the fast spheroidization; (a). Initial specimen; (b). HTC1; (c). HTC2; and (d). HTC3.

The microstructure of the steel SUP9 or 55CrMnA is shown in (Fig. 4) by using the optical microscope with 200x magnification. The production process on the initial specimen of leaf spring steel through the heat treatment process sequence, i.e. hot-rolled, annealing, quenching, and tempering. A microstructural constituent properties identification is required in this experiment [8, 9], the microstructure of the initial specimen Fig. 4 (a) is tempered martensite phase containing carbides distributed in martensite. HTC1 microstructure Fig. 4 (b) is the dissolution of cementite carbide lamellar and forms a fine pearlite phase. The fine pearlite is formed from the effect of a short time in decreasing the austenitization temperature to the temperature of spheroidization. This microstructure was identified as fine cementite that has good characteristic for machining and surface quality. The cooling deceleration of the range 900–723 °C in Fig. 4 (d) makes the percentage increase of the pearlite phase. Where the pearlite phase is identified as an intermetallic compound comprising a mixture lamellar cementite carbide with ferrite constituent [10].

The treatment result showed that the heat treatment condition at 800 °C has good results. The assumption that this is not an effect of austenitization temperature comparison, but resulting from how long time of heating. The cementite lamellar break above the temperature of A_1_ (723 °C) and temperature A_1_ is denoted as the boundary of the calculation in the fast spheroidization, where the austenitization condition is too long will the presence of carbide. In this experiment, the heating time was divided into three conditions; (1) the condition at the spheroidization temperature to austenitization temperature at (723 °C–800 °C), or referred to as heating time ($t_{h}$); (2) the condition while holding time at austenitization temperature ($t_{a}$); and (3) the condition at temperature decrease from holding temperature to spheroidization temperature (800 °C–723 °C) or referred to as cooling time ($t_{c}$), and for magnitude of time of three heating process condition are shown in Table 3.

**Table 3:** The calculation of austenitization heating time.

Heat treatment condition	Time of heating (hour)			Resultant of time
	$t_{h}$	$t_{a}$	$t_{c}$	$t_{r}$
800 °C	0.2265	0.25	0.28	0.756
850 °C	0.3735	0.25	0.462	1.085
900 °C	0.52	0.25	0.644	1.414

The resultant of time (*t*_*r*_) of the temperature 800 °C is 0.756 hours that was achieved from the magnitude of calculation time on the austenitization as expressed in Eq. (1). The holding time of the austenitization temperature 800 °C with holding time 15 minutes as a basic assumption of the subsequent calculation.

(1) $$t_{r}=t_{a}+t_{h}+t_{c}$$

The calculation inspired by the Taguchi method to robust parameter design (RPD) [11, 12], where C. C. Yang et al. [12] used four factors as the parameter to determine the mean value of probability distribution (optimum value). But in this calculation is different with Taguchi method, the mean value of probability distribution has been determined from experiment result i.e. from the resultant time of HTC1 (800 °C), where the parameters of heating and cooling speed were converted to time parameter, the cumulative time of process was denoted as a resultant of time.

(2) $$t_{h}=\left(T_{a}-723\right)/{\dot{T}}_{h}$$

(3) $$t_{c}=\left(T_{a}-723\right)/{\dot{T}}_{c}$$

The temperature of austenitization $\left(T_{a}\right)$ at 10% above the resultant of time (*t*_*r*_) at 800 °C has obtained the resultant time 0.8316 hour and the austenitization temperature at 811.4 °C in same the austenitization holding time ($t_{a})$ at 0.25 hour when condition 10% below of resultant of time ($t_{r})$ was obtained temperature at 788.4 °C.

(4) $$T_{a}=\frac{\left(t_{r}-t_{a}\right){\dot{T}}_{h}{\dot{T}}_{c}}{{\dot{T}}_{h}+{\dot{T}}_{c}}+723$$

**Fig. 5:**
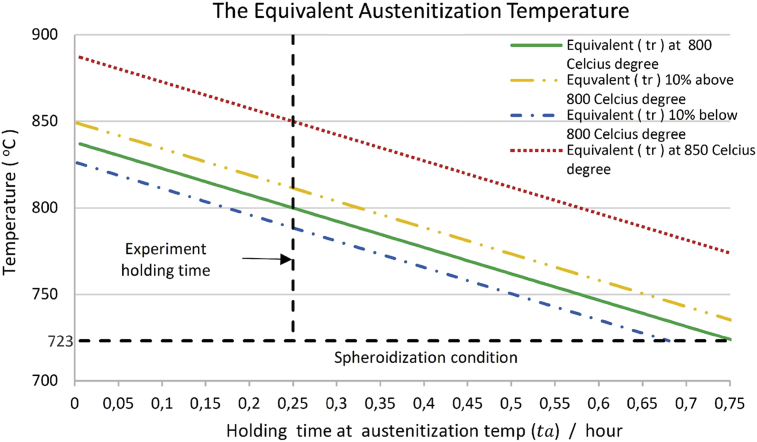
The equivalent Austenitization temperature to resultant of time (*t*_*r*_).

As the result of prediction, the distribution temperature of austenitization able shown in Fig. 5. The figure explained mean value i.e. the experiment holding time at 800 °C used as optimum value to predict the probability distribution in next investigation.

In fact, on the drilling process, the initial workpiece was not able to drill the workpiece, where it blunted caused by wear on the chisel edges. Several investigation declare that is due to an unbalanced axial force with drill diameter [13] or drill bit unable to drill caused by abrasive carbide [14, 15]. The imbalance condition between the twist drill diameter and the axial force does not affect the blunt drill in this investigation, where the specimen of HTC1 (800 °C) the drill bit worked better on the same axial force. The drill bit on HTC1 was able to drill the specimen caused the abrasive carbide transforms into the fine cementite lamellar.

**Fig. 6:**
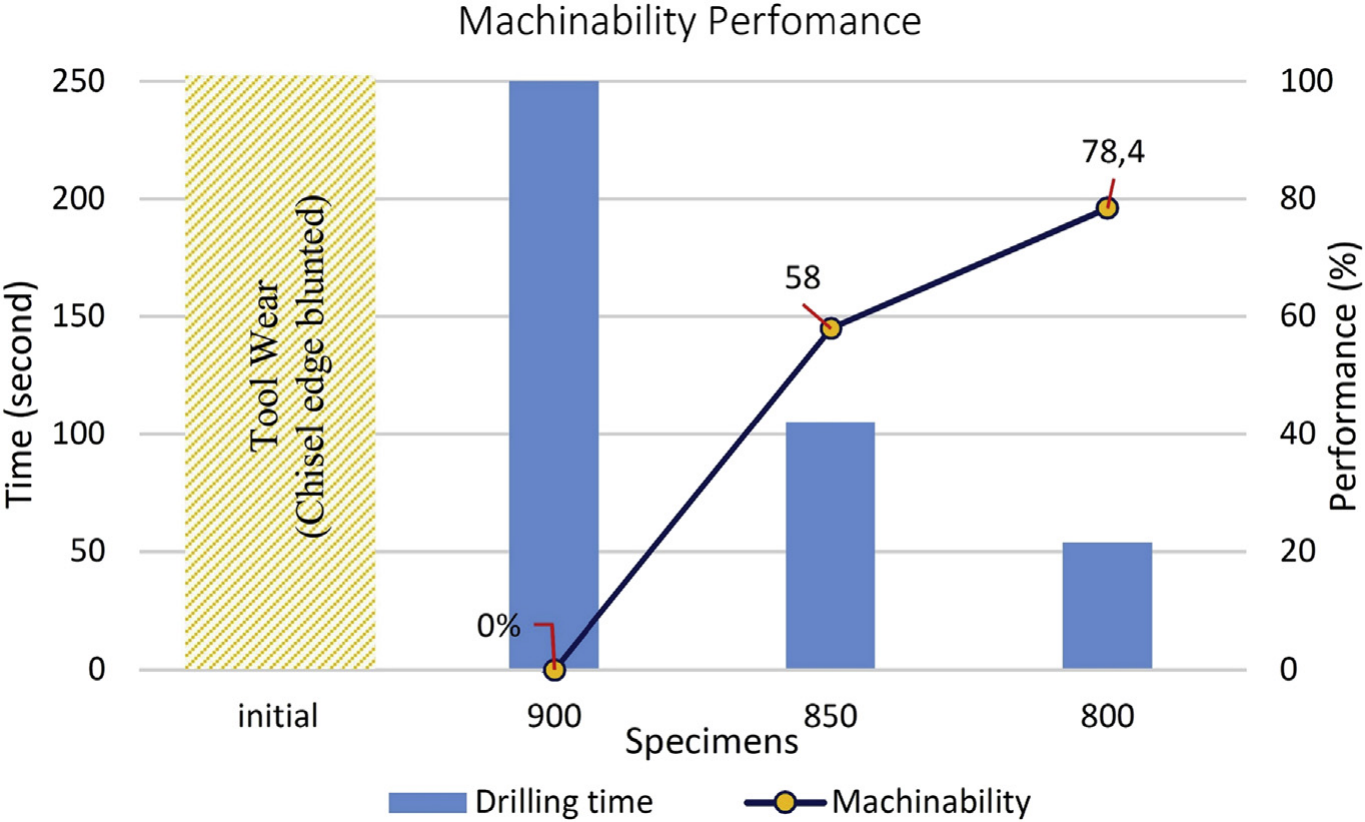
Machinability performance about drilling process time.

Machinability able to define as condition tool lower from wear, generated better surface or less power is needed in machining process. As describe in Fig. 6, tool wear and machining decrease after treatment process. The blunted tool in drilling process of the initial specimen approved that the tool occurred wear, and machining time described in machinability power. The less power was shown in specimen at HTC1 (800 °C), where the time machining is needed at 54 second to able drill specimens, this value was compared with specimen HTC3 (900 °C) to achieved machinability performance.

## 4 Conclusions

Based on the results of the investigation, the high-temperature quality in spheroidization SUP9 spring steel process showed the significant effect on machinability that was occurred in HTC1(800 °C). The distinction in drilling time on the HTC1 (800 °C) with the initial condition, which was revealed by an increase in machinability of 78.4% (54 seconds) against HTC3 drilling time (250 seconds).

The microstructural phase transformation of HTC1 (800 °C) from the abrasive carbide turns into a fine lamellar cementite, which indicated a change in the hardness value of 412 VHN decreasing to 180 VHN. This condition also affects the machinability, where the machining process of the initial specimen, the drill bit was not able to drill caused chisel edge blunted. Instead, the drill bit was able to drill the HTC1 (800 °C) specimen well.

## Declarations

### Author contribution statement

Hendri Chandra and Diah Kusuma Pratiwi: Analyzed and interpreted the data; Wrote the paper.

Muhammad Zahir: Performed the experiments; Wrote the paper.

### Funding statement

This work was supported by the Department of Mechanical Engineering, Sriwijaya University.

### Competing interest statement

The authors declare no conflict of interest.

### Additional information

No additional information is available for this paper.
